# Multiple Introductions of *Yersinia pestis* during Urban Pneumonic Plague Epidemic, Madagascar, 2017

**DOI:** 10.3201/eid3002.230759

**Published:** 2024-02

**Authors:** Voahangy Andrianaivoarimanana, Cyril Savin, Dawn N. Birdsell, Amy J. Vogler, Anne-Sophie Le Guern, Soloandry Rahajandraibe, Sylvie Brémont, Soanandrasana Rahelinirina, Jason W. Sahl, Beza Ramasindrazana, Rado Jean Luc Rakotonanahary, Fanjasoa Rakotomanana, Rindra Randremanana, Viviane Maheriniaina, Vaoary Razafimbia, Aurelia Kwasiborski, Charlotte Balière, Maherisoa Ratsitorahina, Laurence Baril, Paul Keim, Valérie Caro, Voahangy Rasolofo, André Spiegel, Javier Pizarro-Cerda, David M. Wagner, Minoarisoa Rajerison

**Affiliations:** Institut Pasteur de Madagascar, Antananarivo, Madagascar (V. Andrianaivoarimanana, S. Rahelinirina, B. Ramasindrazana, R.J.L. Rakotonanahary, F. Rakotomanana, R. Randremanana, M. Ratsitorahina, L. Baril, V. Rasolofo, A. Spiegel, M. Rajerison);; Institut Pasteur, Paris, France (C. Savin, A.-S. Le Guern, S. Brémont, A. Kwasiborski, C. Balière, V. Caro, J. Pizarro-Cerda);; Northern Arizona University, Flagstaff, Arizona, USA (D.N. Birdsell, A.J. Vogler, J.W. Sahl, P. Keim, D.M. Wagner);; Madagascar Ministry of Public Health, Antananarivo (S. Rahajandraibe, V. Maheriniaina, V. Razafimbia)

**Keywords:** pneumonic plague, *Yersinia pestis*, bacteria, respiratory infections, Madagascar

## Abstract

Pneumonic plague (PP) is characterized by high infection rate, person-to-person transmission, and rapid progression to severe disease. In 2017, a PP epidemic occurred in 2 Madagascar urban areas, Antananarivo and Toamasina. We used epidemiologic data and *Yersinia pestis* genomic characterization to determine the sources of this epidemic. Human plague emerged independently from environmental reservoirs in rural endemic foci >20 times during August–November 2017. Confirmed cases from 5 emergences, including 4 PP cases, were documented in urban areas. Epidemiologic and genetic analyses of cases associated with the first emergence event to reach urban areas confirmed that transmission started in August; spread to Antananarivo, Toamasina, and other locations; and persisted in Antananarivo until at least mid-November. Two other *Y. pestis* lineages may have caused persistent PP transmission chains in Antananarivo. Multiple *Y. pestis* lineages were independently introduced to urban areas from several rural foci via travel of infected persons during the epidemic.

Madagascar reports more human plague (causative agent: *Yersinia pestis*) cases annually than any other country (*1*), often several hundred each season (typically September–March) (*2*). *Y. pestis* persists in multiple rural foci in the central and northern highlands of Madagascar (regions >800 m elevation), wherein it cycles primarily among nonnative rat hosts via nonnative and native flea vectors (*3*,*4*). Occurrence and seasonality of human plague is closely tied to rice cultivation in rural areas (*3*,*4*), which increases contact between humans and rats carrying *Y. pestis*–infected fleas. Most human cases in Madagascar are bubonic plague (BP), which originates from a flea bite (*2*). Fleaborne transmission of *Y. pestis* between humans has not been documented in Madagascar, so all BP cases are considered independently acquired from the environment. Pneumonic plague (PP) is not obtained from the environment but results from untreated BP that progresses to secondary PP (SPP), which subsequently can be passed human-to-human as primary PP. Increases in Madagascar in the proportion of BP cases progressing to PP is attributed to the deteriorating healthcare system (*2*). Human-to-human transmission of PP occurs in Madagascar (*4**–**8*) but is less common. Human plague in urban areas of Madagascar is rare because the rodents and fleas in those areas seldom carry *Y. pestis*.

*Y. pestis* was introduced to Madagascar in 1898, during the third plague pandemic (*9*). Phylogeographic analyses of *Y. pestis* have identified multiple distinct subgroups that occur and persist in specific geographic locations in Madagascar; subgroups are occasionally dispersed between rural foci but rarely become established (*10**–**12*). Given this high fidelity between specific *Y. pestis* molecular subgroups and particular geographic locations in Madagascar, assigning isolates to known molecular subgroups can identify *Y. pestis* dispersal events and likely geographic areas where BP cases were acquired from the environment (*10*).

The 2017–18 human plague season in Madagascar was characterized by a typical number of suspected BP and PP human cases reported from rural endemic foci but an atypically large number of suspected cases, primarily PP, reported from Antananarivo, the capital city, and Toamasina, the main seaport, which are the largest urban areas in Madagascar. We noted a dramatic increase in notified PP cases starting in September 2017, continuing until this urban PP epidemic was officially declared over on November 27, 2017 (*13*). The true number of infections associated with this event remains unknown, as well as whether this urban PP epidemic was caused by an extended chain of transmission of a single clone of *Y. pestis* or by multiple independent introduction events from endemic rural foci (*14*). We prepared detailed case histories for the first documented transmission chain (29 cases) from the PP epidemic and used molecular characterization of *Y. pestis* isolates and human sputum samples obtained from urban areas and rural endemic foci in 2017 to determine the sources of this urban PP epidemic.

## Methods

### Definitions and Investigation

In August–December 2017, plague cases in Madagascar were notified to the plague national surveillance system, which is mandatory; no ethics approval is required to use those public health data. We classified cases as suspect, probable, or confirmed as previously defined (*13*) and as urban if they occurred within cities with population >150,000. We considered disease onset as the first day plague symptoms occurred. We conducted an epidemiologic description for the 29 initial human cases. We have anonymized information for all cases.

### Sample Analysis

*Y. pestis* was isolated from human samples and tested for susceptibility to multiple antimicrobial drugs as previously described (Appendix 1 sections 1, 2) (*13*); we generated whole-genome sequences (WGSs) for isolates (Appendix 1 section 3, Table 1). We conducted 2 rounds of targeted capture and enrichment of *Y. pestis* DNA from DNA extracted from sputum samples positive for *Y. pestis* via PCR, and then sequenced the enriched samples (Appendix 1 sections 5,6, Table 2). We inferred a maximum-likelihood phylogeny using single nucleotide polymorphisms (SNPs) identified from 36 WGSs from 2017 isolates and 54 other isolates representative of the overall phylogenetic diversity of *Y. pestis* in Madagascar (*10*) (Appendix 1 section 4, Table 1; Appendix 2). SNPs identified in the phylogeny as specific to 2017 isolates, or to clades containing 2017 isolates, were queried in sequence data from enriched sputum samples (Appendix 1, section 7, Table 2).

### Emergence Events

We determined that multiple 2017 human isolates resulted from the same emergence of *Y. pestis* from environmental reservoirs into humans if they differed by <2 SNPs in the phylogeny. We defined an emergence as independent from other emergences if there was no epidemiologic association and if the isolates from that emergence were more closely related to older isolates than to other 2017 emergences or differed from other 2017 emergences by >5 SNPs.

## Results

### Cases, Isolates, and Samples

A total of 2,549 suspected plague cases were notified throughout Madagascar during August–December 2017, including 1,347 from urban areas and 1,241 classified as PP. Confirmed or probable PP cases were reported from Antananarivo during August 28–November 20 and from Toamasina during September 12–October 27, 2017. We obtained and sequenced *Y. pestis* isolates from 36 cases from 2017 (Appendix 1 sections 1, 3, Table 1). We identified SNPs specific to 2017 isolates or to clades containing 2017 isolates in 7 enriched sputum samples (Appendix 1 section 7, Table 5).

### Multiple Emergences of Human Plague in Rural Endemic Foci

**Figure 1:**
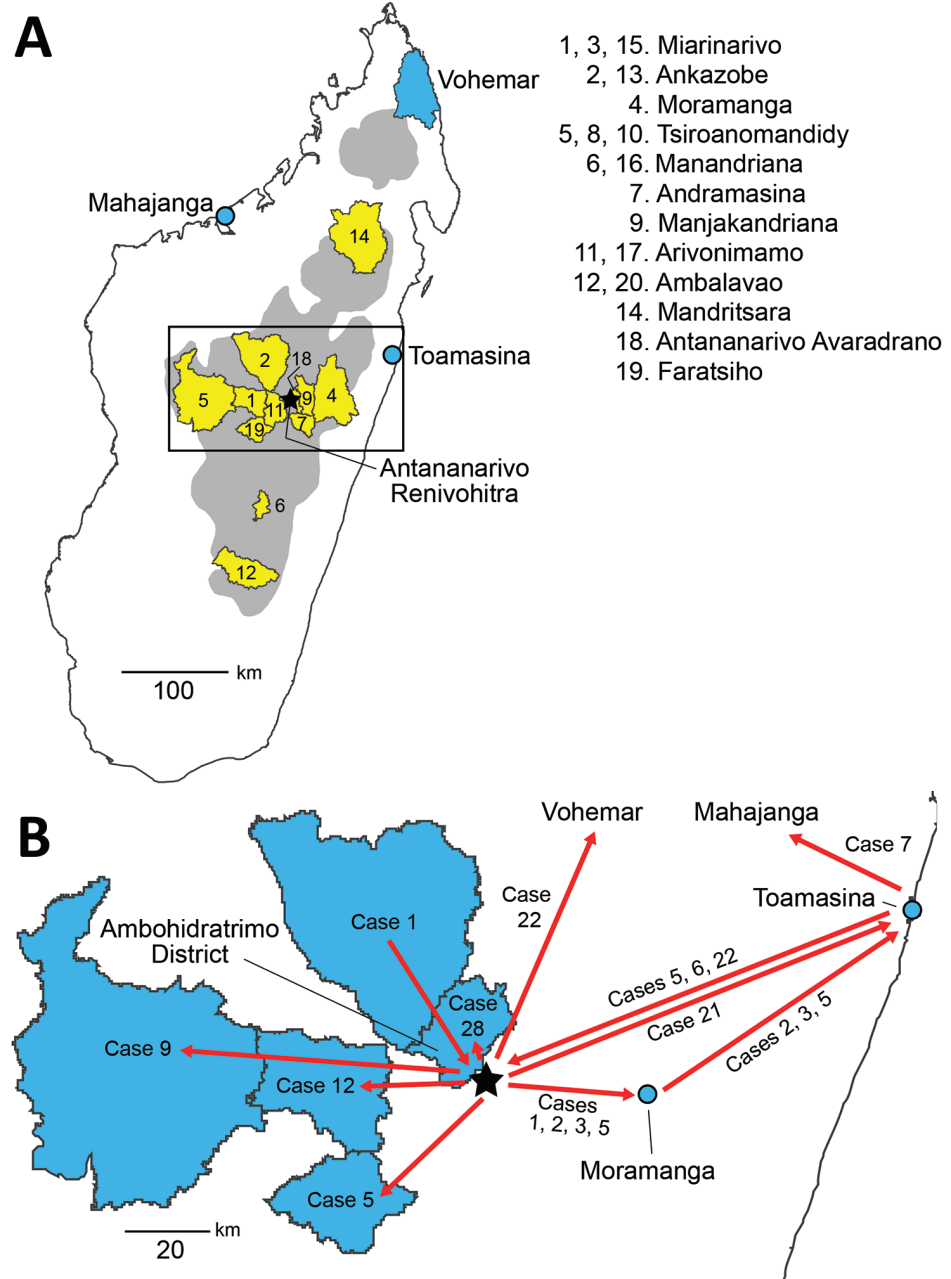
Plague emergence in Madagascar, August–November 2017. A) Locations of emergences. Gray shading indicates plague-endemic regions in the central and northern highlands; yellow shading indicates 12 districts from which human plague emerged from environmental reservoirs 20 times during August–November 2017. Districts are listed in chronological order of emergences. Multiple numbers in the list correspond to different independent emergences from the same district (Table 1); only the first number is indicated on the map. Black box indicates the area shown in panel B. B) Movements (red arrows) of some of the cases (Table 2) associated with the first urban pneumonic plague transmission chain (emergence 2 in Table 1). Blue polygons indicate districts of origin/destination for travel; blue circles indicate the cities of Moramanga and Toamasina.

**Figure 2:**
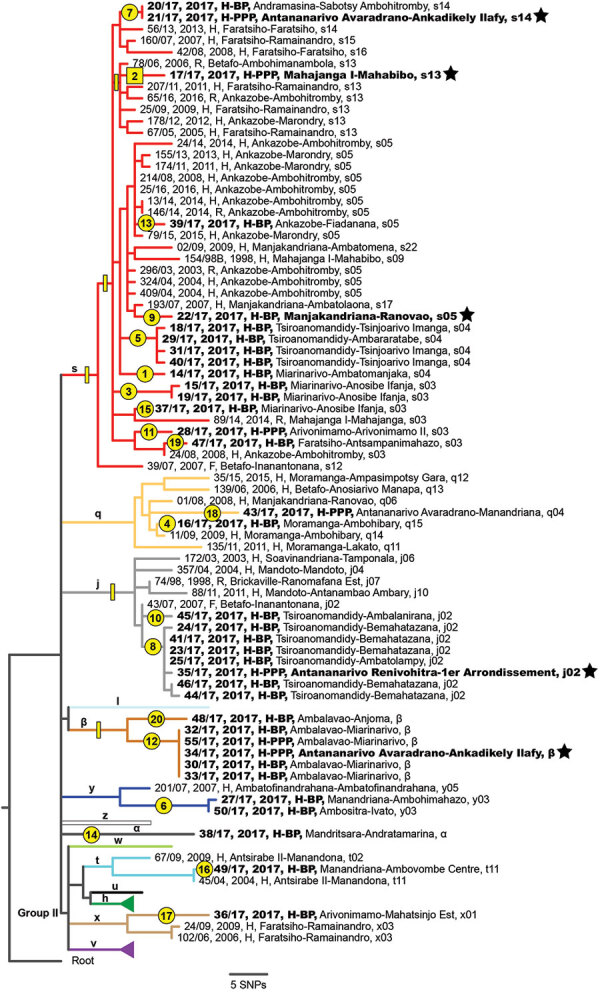
Maximum-likelihood phylogeny of 90 *Yersinia pestis* isolates obtained in rural endemic foci from Madagascar during August–November 2017 (boldface) and reference sequences. Tree was created using 483 core-genome SNPs discovered from WGSs and rooted using North American strain CO92. Stars indicate 5 isolates obtained within the urban areas of Antananarivo or Mahajanga. Numbers in yellow circles and squares indicate 20 emergence events from environmental reservoirs (Table 1); yellow squares and rectangles along branches indicate phylogenetic position of SNPs that were queried in *Y. pestis* sequence data from enriched sputum samples (Appendix 1, https://wwwnc.cdc.gov/EID/article/30/2/23-0759-App1.pdf). Labels for each isolate indicate identification number, year of isolation, host-disease form, and district-commune of isolation; letters on branches and colors of branches indicate known lineages (*10*). Some known lineages without isolates during August–November 2017 are unlabeled or collapsed. BP, bubonic plague; H, human; F, flea; PP, pneumonic plague; PPP, primary pneumonic plague; R, rat; SNP, single-nucleotide polymorphism; WGS, whole-genome sequencing. An expanded figure is available online (https://wwwnc.cdc.gov/EID/article/30/2/23-0759-F2.pdf).

Starting in Miarinarivo District in August, human plague emerged independently from environmental reservoirs >20 times in multiple rural endemic foci in Madagascar during August–November 2017 (Table 1; Appendix 1 section 8). Those events occurred in 19 different communes located in 12 different districts in the central and northern highlands (Table 1; Figure 1). Clinical *Y. pestis* isolates obtained from them were closely related to previous isolates from those locations (Figure 2), confirming *Y. pestis* continues to persist in the environment in these regions (*10*). PP arose from BP in at least 6 of these events; confirmed human cases originating from 5 events, including 4 PP cases, were documented in urban areas during the epidemic (Table 1).

**Table 1:** Twenty emergence events of *Yersinia pestis *bacteria from environmental reservoirs into humans in rural plague foci, Madagascar, August–November 2017

Event	Major *Y. pestis* group	District/Commune	Earliest recorded onset date	Progression from BP to PP?	Travel	Spread to urban areas (evidence)
1	s	Miarinarivo/Ambatomanjaka	Aug 13	No	No	
2	s	Ankazobe/Marondry	Aug 25	Yes	Yes	Toamasina (sputum 135–2017), Mahajanga (isolate 17/17), Antananarivo (sputum 2093–2017)
3	s	Miarinarivo/Anosibe Ifanja	Aug 26	No	No	
4	q	Moramanga/Ambohibary	Sep 2	No	No	
5	s	Tsiroanomandidy/Tsinjoarivo Imanga	Sep 16	No	No	
6	y	Manandriana/Ambohimahazo	Sep 17	No	No	
7	s	Andramasina/Sabotsy Ambohitromby	Sep 21	Yes	Yes	Antananarivo (isolate 21/17)
8	j	Tsiroanomandidy/Bemahatazana	Sep 26	Yes	Yes	Antananarivo (isolate 35/17, possibly sputum 819–2017)
9	s	Manjakandriana/Ranovao	Oct 2	No	Yes	Antananarivo (isolate 22/17)
10	j	Tsiroanomandidy/Ambalanirana	Oct 3	No	No	Antananarivo (possibly sputum 819–2017)
11	s	Arivonimamo/Arivonimamo II	Oct 5	Yes	No	
12	β	Ambalavao/Miarinarivo	Oct 7	Yes	Yes	Antananarivo (isolate 34/17, sputum 1494–2017)
13	s	Ankazobe/Fiadanana	Oct 8	No	No	
14	α	Mandritsara/Andratamarina	Oct 14	No	No	
15	s	Miarinarivo/Anosibe Ifanja	Oct 17	No	No	
16	t	Manandriana/Ambovombe Centre	Oct 18	No	No	
17	x	Arivonimamo/Mahatsinjo Est	Oct 23	No	No	
18	q	Antananarivo Avaradrano/Manandriana	Oct 30	Yes	Yes	
19	s	Faratsiho/Antsampanimahazo	Nov 7	No	No	
20	β	Ambalavao/Anjoma	Nov 9	No	No	

### Initial 29 Cases Associated with the First Urban PP Transmission Chain

**Figure 3:**
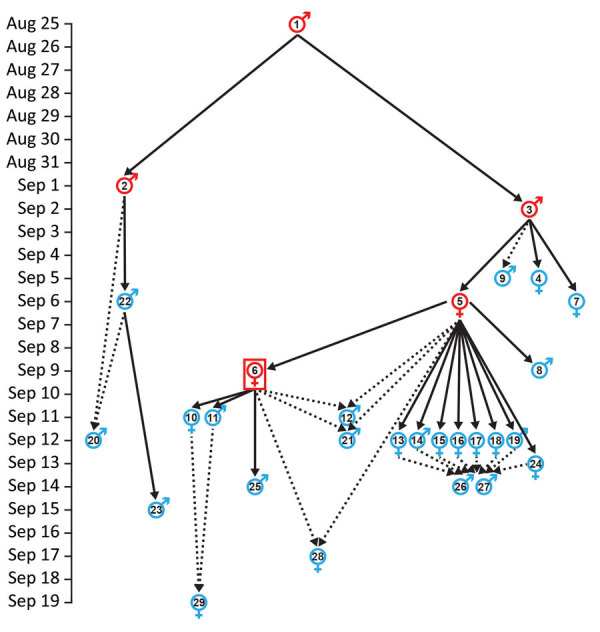
Transmission patterns among the initial 29 cases associated with the first urban PP transmission chain in Madagascar, August–November 2017 (emergence 2 in Table 1). Dates are illness onset dates. Solid arrows indicate likely infection sources based on known contact (Table 2); dotted arrows indicate hypothetical infection sources inferred from genetic data, epidemiologic data, or both. Symbols for individual cases indicate male or female sex; red indicates persons who died and blue indicates survivors. Red box indicates the first identified case from the epidemic that triggered the subsequent public health response.

Case-patient 1 exhibited PP symptoms (fever, gastrointestinal and respiratory distress, but no cough) on August 25, 2017, in Ankazobe District (Tables 1, 2; Figures 1, 3) where he had been living and working and elected to travel to his permanent home in Toamasina. His employer drove him by car to Antananarivo, where he boarded a shared bush taxi to Toamasina on August 27; case-patient 2 sat beside him; case-patient 3 immediately behind; and case-patient 5, wife of case-patient 3, beside her spouse. That same day near Moramanga, case-patient 1 experienced a deteriorated health status, including respiratory distress, and died; case-patients 2 and 3 cared for him while he was dying. The corpse was removed at the Moramanga health center, and the bush taxi continued to Toamasina.

**Table 2:** Information on 29 case-patients associated with the first known urban pneumonic plague transmission chain, Madagascar, August–November 2017*

Case-patient no.	Age, y/sex	Outcome	Onset date	Onset district	Onset location	Travel	Documented contact with other case-patients	Sample/positive *Y. pestis* result	Case definition
1	32/M	Died	Aug 25	Ankazobe	Rural	Yes	2, 3, 5	No samples collected	Suspected
2	26/M	Died	Sep 1	Toamasina I	Urban	Yes	1, 3, 5, 20, 22	No samples collected	Suspected
3	36/M	Died	Sep 2	Toamasina I	Urban	Yes	1, 2, 4, 5, 7	No samples collected	Suspected
4	23/F	Recovered	Sep 5	Toamasina I	Urban	No	3, 5, 7	Sputum 135–2017/RDT, PCR	Probable
5	16/F	Died	Sep 6	Antananarivo-Renivohitra	Urban	Yes	1, 3, 4, 6, 7, 8, 9, 12, 13, 14, 15, 16, 17, 18, 19, 21, 24, 28	No samples collected	Suspected
6	47/F	Died	Sep 9	Antananarivo-Renivohitra	Urban	Yes	5, 8, 9, 10, 11, 12, 21, 25, 28, 29	Sputum 118–2017/RDT, PCR	Probable
7	40/F	Recovered	Sep 6	Mahajanga I	Urban	Yes	3, 4, 5	Sputum 121–2017/culture yielded isolate 17/17	Confirmed
8	45/M	Recovered	Sep 9	Antananarivo-Renivohitra	Urban	No	5, 6, 9, 12, 21, 28	Sputum 150–2017/RDT	Probable
9	30/M	Recovered	Sep 5	Tsiroanomandidy	Rural	Yes	5, 6, 8, 12, 21, 28	Sputum 143–2017/RDT	Probable
10	21/F	Recovered	Sep 11	Antananarivo-Renivohitra	Urban	No	6, 11, 29	Sputum 119–2017/no positive tests	Suspect
11	15/M	Recovered	Sep 11	Antananarivo-Renivohitra	Urban	No	6, 10, 29	Sputum 120–2017/no positive tests	Suspected
12	37/M	Recovered	Sep 11	Miarinarivo	Rural	Yes	5, 6, 8, 9, 21, 28	Sputum 153–2017/RDT	Probable
13	11/F	Recovered	Sep 12	Faratsiho	Rural	No	5, 14, 15, 16, 17, 18, 19, 24	No samples collected	Suspected
14	52/M	Recovered	Sep 12	Faratsiho	Rural	No	5, 13, 15, 16, 17, 18, 19, 24	Sputum 124–2017/RDT, PCR	Probable
15	48/F	Recovered	Sep 12	Faratsiho	Rural	No	5, 13, 14, 16, 17, 18, 19, 24	Sputum 125–2017/RDT, PCR	Probable
16	9/F	Recovered	Sep 12	Faratsiho	Rural	No	5, 13, 14, 15, 17, 18, 19, 24	No samples collected	Suspected
17	39/F	Recovered	Sep 12	Faratsiho	Rural	No	5, 13, 14, 15, 16, 18, 19, 24	No samples collected	Suspected
18	38/F	Recovered	Sep 12	Faratsiho	Rural	No	5, 13, 14, 15, 16, 17, 19, 24	Sputum 129–2017/RDT	Probable
19	19/M	Recovered	Sep 12	Faratsiho	Rural	No	5, 13, 14, 15, 16, 17, 18, 24	No samples collected	Suspected
20	25/M	Recovered	Sep 12	Toamasina I	Urban	No	2	Sputum 136–2017/RDT	Probable
21	31/M	Recovered	Sep 12	Toamasina I	Urban	Yes	5, 6, 8, 9, 12, 28	Sputum 149–2017/RDT, PCR	Probable
22	30/M	Recovered	Sep 6	Vohemar	Rural	Yes	2, 23	Sputum 184–2017/RDT	Probable
23	33/M	Recovered	Sep 15	Vohemar	Rural	No	22	Sputum 185–2017/RDT	Probable
24	UNK/F	Recovered	Sep 13	Faratsiho	Rural	No	5, 13, 14, 15, 16, 17, 18, 19	Sputum 131–2017/no positive tests	Suspected
25	49/M	Recovered	Sep 14	Antananarivo-Renivohitra	Urban	No	6	Blood 188–2017/RDT	Probable
26	13/M	Recovered	Sep 14	Faratsiho	Rural	No	≥1 of 13, 14, 15, 16, 17, 18, 19, 24	Sputum 133–2017/RDT, PCR	Probable
27	39/M	Recovered	Sep 14	Faratsiho	Rural	No	≥1 of 13, 14, 15, 16, 17, 18, 19, 24	Sputum 134–2017/no positive tests	Suspected
28	25/F	Recovered	Sep 17	Ambohidratrimo	Rural	Yes	5, 6, 8, 9, 12, 21	Sputum 164–2017/no positive tests	Suspected
29	46/F	Recovered	Sep 10	Antananarivo-Renivohitra	Urban	No	6, 10, 11	Sputum 181–2017/RDT	Probable

*UNK, unknown.

Case-patient 2 experienced PP symptoms in Toamasina on September 1 and died there September 2. His brother (case-patient 22) transferred the corpse by car from Toamasina to Antananarivo, by plane from Antananarivo to Sambava-Vohemar, and then by car and on foot to their native village at Ambodisakoa, Vohemar District, where case-patient 2 was buried on September 6. Case-patient 22 sought care for PP symptoms on September 6 and was admitted to a hospital at Vohemar, where he recovered. Case-patient 23, brother-in-law of case-patient 2, sought care for PP symptoms at Vohemar on September 15 and was successfully treated. No other cases were reported from Vohemar. Case-patient 20, a friend of case-patient 2 who had direct contact with him during his disease, was admitted to a hospital at Toamasina with PP symptoms on September 12; case-patient 2 might have also had contact with case-patient 22 in Toamasina.

Case-patient 3 was admitted to hospital at Toamasina with PP symptoms on September 2 and died on September 3; a wake was held that night at Toamasina. Case-patients 5 and 7 (sister of case-patient 3, resident of Mahajanga) attended the wake. Case-patient 4, a nurse who cared for case-patient 3, exhibited PP symptoms on September 5 and fully recovered after treatment; no secondary cases were identified from case-patient 4. Case-patient 7 returned to Mahajanga, where she experienced PP symptoms on September 6; she fully recovered after treatment and no additional cases were reported from Mahajanga.

On September 4, case-patients 5 and 6 (another sister of case-patient 3) transferred the corpse of case-patient 3 by car from Toamasina to Antohomadinika-Antananarivo, where another night wake was held from September 4–6, followed by burial in Andramasina on September 6. Case-patients 5, 6, 8, 9, 12, 21, and 28 attended the night wake, funeral, or both; case-patient 5 exhibited PP symptoms on September 6. Case-patient 8 exhibited PP symptoms in Antananarivo on September 9, case-patient 9 in Tsiroanomandidy District on September 5, case-patient 12 in Miarinarivo District on September 11, case-patient 21 in Toamasina on September 12, and case-patient 28 in Ambohidratrimo District on September 17. Case-patients 8, 9, 12, 21, and 28 recovered after treatment, and no secondary cases were reported from them.

Case-patient 5 started travel to Faratsiho on September 9 but died on the way. It is unknown who was traveling with her. Her corpse was transported to Alatsinainy Bandroka, Faratsiho District, and buried after a traditional funeral. Case-patients 13–19 and 24, all Faratsiho residents, helped prepare the corpse, participated in the funeral, or both; all exhibited PP symptoms starting on September 12 (case-patients 13–19) or 13 (case-patient 24). Case-patients 26 and 27 had documented but unspecified contacts with >1 of the above cases and exhibited PP symptoms on September 14. All case-patients in Faratsiho recovered after treatment.

Case-patient 6 experienced PP symptoms on September 9 in Antananarivo, sought care at a private clinic with a physically altered state on September 11, and was transferred to a military hospital where she died on September 11; this case was the first identified case from the epidemic that triggered the subsequent public health response (*13*). Four secondary cases in Antananarivo resulted from contact with case-patient 6: case-patients 10 and 11 (daughter and son of case patient 6; PP onset September 11), case-patient 29 (daughter-in-law of case-patient 6; PP onset September 19), and case-patient 25. Case-patient 25 (onset September 14), who had septicemic plague, was a health agent and handled and disinfected the corpse of case-patient 6.

Sample 121–2017, collected in Mahajanga from case-patient 7, yielded the only *Y. pestis* isolate obtained from this transmission chain, 17/17, which is most closely related to older isolates from Ankazobe, Faratsiho, and Betafo districts (Figure 2). This result is consistent with Ankazobe District as the geographic source of this transmission chain; case-patient 1 was living and working there but had no recent travel to Faratsiho or Betafo Districts. Enrichment and sequencing of sputum samples obtained from case-patient 4 in Toamasina (135–2017), case-patient 15 in Faratsiho (125–2017), and case-patient 22 in Vohemar (184–2017) (Table 2) revealed the presence in those samples of 1–5 SNPs specific to isolate 17/17 (Appendix 1 section 7, Table 5), documenting that those cases were all part of a single transmission chain that was spread to multiple urban areas and regions in Madagascar. On November 8, sputum sample 2093–2017 was collected from an Antananarivo resident with no recent travel outside the city (Table 1). After we enriched and sequenced this sample, we determined that it contained 3 SNPs specific to isolate 17/17 that were also present in sputum sample 125–2017 from case-patient 15 (Appendix 1 section 7, Table 5), suggesting possible community spread of this transmission chain in Antananarivo and persistence there until at least November 2017.

### Introduction of Other *Y. pestis* Lineages to Antananarivo

Isolate 21/17 was obtained from a sputum sample collected on September 29 from an Antananarivo resident. It is distinct from isolate 17/17 but identical to isolate 20/17 (Figure 2), which was obtained from a bubo aspirate collected on September 22 from a resident of a rural area of Andramasina District (Table 1; Appendix 1 section 8). There was no known relationship between those 2 persons, and neither reported travel. The person yielding isolate 20/17 had clinical signs consistent with SPP, including cough for <5 days. Although unknown, this person may have initiated an undocumented PP outbreak in Andramasina District; another person infected from that outbreak may have traveled to Antananarivo, leading to a transmission chain there that infected the person who yielded 21/17.

A foreign tourist sought care at a hospital in Antananarivo on October 1 for PP symptoms; he had traveled outside Antananarivo but the specific details are unknown. His sputum sample yielded isolate 35/17, which is distinct from isolate 17/17 but closely related to multiple human isolates obtained from BP cases in Tsiroanomandidy District starting in September (Table 1; Figure 2). This finding suggests plague activity in this rural focus as the ultimate source of the infection in the tourist, from whom no known secondary cases were reported. However, sequencing of enriched sputum sample 819–2017, collected on October 12 from an Antananarivo resident with no recent travel, contained 1 SNP specific to the j phylogenetic group of *Y. pestis* in Madagascar (Appendix 1 section 7, Table 5), which suggested the possibility of additional undocumented cases associated with this transmission chain in Antananarivo. Emergence 10 was also assigned to phylogenetic group j (Table 1; Figure 2), so it is possible the patient who yielded sputum sample 819–2017 could have been infected through a transmission chain from that event involving undocumented travel to Antananarivo.

Isolate 22/17 (Table 1) was obtained on October 2 from a bubo aspirate collected in Antananarivo from a girl who died the next day. She resided in a rural region of Manjakandriana District; her family brought her to Antananarivo for treatment when she became ill. Upon sequencing, isolate 22/17 was distinct from isolate 17/17 and most closely related to a 2007 human isolate from Manjakandriana District (Figure 2). No secondary cases were reported from this person, which is not unexpected because it was BP.

Isolate 34/17 was reportedly collected from a newborn baby in Antananarivo on October 15; his parents were residents of Antananarivo with no recent travel. The baby was febrile and transferred to a children’s hospital as a suspect plague case; staff collected a sputum sample using a bronchoscope. The sputum sample attributed to this newborn yielded isolate 34/17, which is distinct from isolate 17/17 but identical to multiple human isolates obtained from plague activity in a rural region of Ambalavao District (Table 1; Figure 2; Appendix 1 section 8). The earliest documented case there (onset date October 7) yielded isolate 32/17 and was diagnosed as BP, but PP symptoms were also present. Subsequent PP cases were reported from this rural area into late November. No secondary cases were reported from the baby, but SNP data suggest subsequent community spread of closely related *Y. pestis* in Antananarivo. 

On October 24, sputum sample 1494–2017 was collected from an Antananarivo resident with no travel history. Enrichment and sequencing of that sample determined it contained an SNP specific to human isolates 30/17, 32/17, 33/17, 34/17, and 55/17 obtained in Ambalavao District as part of the investigation of emergence 12 (Appendix 1 section 8).

## Discussion

Our epidemiologic and genomic analyses of 29 human plague cases associated with the first emergence event to reach urban areas of Madagascar document that the transmission chain started in late August 2017 in rural Ankazobe District and subsequently spread to >8 other districts by mid-September, including the urban areas of Antananarivo, Toamasina, and Mahajanga, as well as multiple rural districts (Table 2; Figure 1). This transmission chain demonstrates that movements of infected persons can rapidly spread PP across large distances. Prompt public health responses prevented subsequent spread of this transmission chain in Mahajanga and Vohemar, but it apparently persisted into mid-November 2017 in Antananarivo. Although we did not identify genetic data to confirm continued presence of this transmission chain in Toamasina, it is located well outside the plague-endemic region in Madagascar; confirmed and probable PP cases continued to be reported there through mid-October, which suggests possible community spread. Similar to previous reports from Madagascar and elsewhere in Africa (*4*,*6**–**8*,*15**–**17*), this PP transmission chain was associated with traditional funeral practices.

PP transmission requires close contact with an infected person in the end stage of disease (*14*,*18*). On the day he died, case-patient 1 traveled both in a car with his employer and in a bush taxi. Bush taxis in Madagascar are extremely crowded environments, typically loaded beyond the original capacity of the vehicle and departing only when completely full (*19*). Despite this close contact, only 2 secondary cases, case-patients 2 and 3 (Figure 1), appear to have resulted directly from the presence of case-patient 1 in the bush taxi and none from travel in the car. This result is likely because of his lack of cough; transmission of PP is thought to occur from either inhalation of respiratory droplets expelled by coughing persons or by direct contact (*14*,*18*), and only those 2 secondary cases were known to have had direct contact with case-patient 1 in the bush taxi.

Assessing the true number of plague cases associated with the 2017 urban PP epidemic in Madagascar has been challenging for multiple reasons (*13*,*20*). One reason is that sputum is a poor-quality sample type; isolating *Y. pestis* from sputum is always complicated by commensal flora and sample quality, and the large number of sputum samples collected during this epidemic overwhelmed public health laboratories (*13*). The associated delay in culturing sputum samples, as well as evidence that many patients may have been self-administering antimicrobial drugs effective against *Y. pestis* (*13*,*20*), likely further explains why so few *Y. pestis* isolates were obtained from this epidemic. It is also possible that many of the suspected and probable cases were not true infections (*14*,*21*). Regardless, only 4 isolates were obtained from sputum samples collected from urban areas during this epidemic: 17/17 from Mahajanga and 35/17, 21/17, and 34/17 from Antananarivo. Isolate 22/17 also was collected in Antananarivo from a BP case. All 5 of the isolates obtained in urban areas are highly distinct from each other (Figure 2) and associated with 5 different independent emergence events in rural foci (Table 1). Those patterns document that *Y. pestis* was introduced to Antananarivo >5 times during the epidemic, with evidence that 3 of those introductions may have led to subsequent community transmission. The patterns also suggest there may have been more introductions of *Y. pestis* to Antananarivo, Toamasina, or both that could not be documented because there were no isolates.

Movement of infected persons from rural disease-endemic regions to urban areas may have been caused by panic that arose in the Malagasy population in response to the PP epidemic (*1*). The number of notified cases associated with this epidemic increased dramatically in late September and early October 2017 (*13*), coinciding with the timing of the first situation report from the World Health Organization (*22*) and widespread coverage in the media; this increase in cases was probably caused in part from fear and panic in urban inhabitants unfamiliar with plague (*20*). Residents of rural regions are much more aware of plague and perceive it to be rapidly fatal; however, they are less familiar with PP than BP (*23*). The increased public communications regarding the PP epidemic in urban areas, as well as the increased availability there of public health resources from mobilization of substantial domestic and international public health resources (*1*), may have led some infected persons from rural areas to travel to Antananarivo to seek treatment. A previous study (*11*) described evidence of plague-infected persons moving from the central highlands to Mahajanga and from Mahajanga to Antananarivo.

In conclusion, the 2017 urban PP epidemic in Madagascar involved the introduction of multiple independent lineages of *Y. pestis* from several rural foci, which may partly explain why this epidemic was so difficult to control. Because PP spreads person-to-person, control of PP outbreaks and epidemics is focused on identifying cases and their known contacts and providing antimicrobial treatment. Those types of investigations were largely impossible in this instance, given the extent of the epidemic, which likely hampered control efforts. Our results suggest that control efforts also might have been diminished by the presence of multiple independent transmission chains that may have resulted in continuation or expansion of the epidemic. Our findings highlight the importance of using existing genotyping tools (*24*) and developing genomics capabilities in Madagascar, elsewhere in Africa, and other global locations (*25*) so they can be used during outbreaks of plague and other diseases to promptly identify multiple sources and transmission chains to better inform control efforts.

Appendix 1 Additional information about multiple introductions of *Yersinia pestis* during urban pneumonic plague epidemic, Madagascar, 2017.

Appendix 2 Single nucleotide polymorphisms analyzed in study of *Yersinia pestis* during urban pneumonic plague epidemic, Madagascar, 2017.
