# Supplementary material for: Multiple Introductions of Yersinia pestis during Urban Pneumonic Plague Epidemic, Madagascar, 2017
Source: Emerg Infect Dis. 2024 Feb;30(2):289–98. doi: 10.3201/eid3002.230759 (PMC10826772; doi:10.3201/eid3002.230759)
Supplement: Appendix 1 — Additional information about multiple introductions of Yersinia pestis during urban pneumonic plague epidemic, Madagascar, 2017. [file 23-0759-Techapp-s1.pdf]

Article DOI: http://doi.org/10.3201/eid3002.230759

EID cannot ensure accessibility for supplementary materials supplied by authors. Readers who have difficulty accessing supplementary content should contact the authors for assistance.

Multiple Introductions of *Yersinia pestis* during Urban Pneumonic Plague Epidemic, Madagascar, 2017

Appendix

1. Collection and Testing of Clinical Samples

Biologic samples (bubo, sputum, blood, liver, and lung biopsies) from clinically suspected plague patients were sent to the Malagasy Central Laboratory for Plague (CLP) housed at the Plague Unit of the Institut Pasteur de Madagascar (IPM) for biologic confirmation as part of Madagascar's national plague surveillance program overseen by the Malagasy Ministry of Health. This program requires declaration of all suspected human plague cases and collection of biologic samples from those cases. These samples and any cultures or DNA derived from those samples are all delinked from the patients from whom they originated and are analyzed anonymously if used in any research study, such as this one. As such, additional review of this research was not required due to the anonymous nature of the samples. Storage conditions for samples varied before receipt at IPM, but once at IPM all samples were at −20°C. A rapid diagnostic test (RDT) against F1-antigen (*1*) (produced by IPM) was performed on samples from most suspect cases, and DNA was extracted from many samples and tested with PCR, as previously described (*2*). Each sample also was streaked on selective medium Cefsulodin Irgasan Novobiocin (CIN, Oxoid) agar plates and cultured in peptone broth before incubation at 26°C for more than 48h. In parallel, amplification by intraperitoneal injection of the sample was also performed in two laboratory mice (*Mus musculus*) that were subsequently observed for 10 days; this work was performed in a BSL2 animal facility at IPM. These animal studies were performed in accordance with the directive 2010/63/EU of the European Parliament (http://eur-lex.europa.eu/LexUriServ/LexUriServ.do?uri=OJ:L:2010:276:0033:0079:EN:PDF). If a mouse died between 2–10 days post injection, the spleen was removed, macerated, and cultured on CIN

agar plate for isolation of *Y. pestis* (*3*). Suspected *Y. pestis* colonies were then enriched on Brain Heart Infusion slant agar media for 24h before biochemical identification using API20E (Biomérieux) strips and bacteriophage lysis test. Isolated *Y. pestis* strains were stored in glycerol conservation media at −80°C and sent on dry ice to the Yersinia Research Unit of the Institut Pasteur in Paris, France for genomic sequencing and analysis.

2. Antimicrobial Testing of Isolates

Each *Y. pestis* isolate was tested for susceptibility to gentamycin, ciprofloxacin, trimethoprim-sulfamethoxazole, chloramphenicol, tetracycline, and streptomycin using a disk diffusion method as per the Clinical Laboratory Standards Institute (CSLI) guidelines, as previously described (*4*). All were susceptible to all tested antimicrobials.

3. Whole-Genome Sequencing of Isolates

Isolates were subcultured in Luria-Bertani broth at 28°C for 48h. Two ml of culture was centrifuged at 6,000 g for 5 minutes. The pellet was then subjected to DNA extraction with PureLinkTM Genomic DNA minikit (K1820–02, Invitrogen) following the manufacturer's instructions except that DNA was eluted using 150 µL of nuclease-free H2O (AM9937, Ambion). Preparation of the libraries for genomic sequencing was performed with TruSeq Nano DNA library HT Prep kit (Illumina, San Diego, California, USA) following the manufacturer's instructions. Library quality was evaluated with Agilent High sensitivity DNA chips (2100 Bioanalyzer, Agilent) and libraries were quantified with KAPA library Quantification kit (Lightcycler®480, Roche). The resulting libraries were strategically clustered to achieve at least 1.5 M of reads per sample and sequenced on an Illumina MiSeq platform for 250bp paired-end sequencing with MiSeq Reagent Kit v2 (500-cycles). Raw sequence data for all strains have been deposited at NCBI under BioProject PRJNA504742; accession numbers for individual strains are provided in Appendix 1 Table 1.

4. SNP Discovery from Isolates and Phylogenetic Methods

Single nucleotide polymorphisms (SNP) were discovered from whole genome sequences of 90 *Y. pestis* isolates from Madagascar; accession numbers and other information are provided

in Appendix 1 Table 1. Raw reads were aligned against reference genome *Y. pestis* CO92 (GCF_000009065.1) (*5*) with minimap2 v2.24 (*6*). SNPs were called with GATK v4.2.6.1 (*7*). SNPs with a depth of coverage <10 or a proportion <0.90 were filtered from downstream analyses. Any position that fell within a duplicated region, based on a reference self-alignment with NUCmer v3.1 (*8*), was also filtered; all methods were wrapped by NASP v1.2.0 (*9*). Additional positions were filtered from the matrix if they were associated with known CO92 errors (*9*), were in close proximity to other SNPs (<5 nt), or were found in tandem repeats annotated with Tandem Repeats Finder v4.10.0 (*10*). A maximum likelihood phylogeny was inferred from a concatenated SNP alignment with IQ-TREE v2.2.0.3 (*11*) using the integrated ModelFinder method (*12*). The final phylogeny was constructed using 483 SNPs (see separate supplemental Excel file [Appendix 2], derived SNP allele states are highlighted in blue in that file), including 455 SNPs that varied among the 90 *Y. pestis* isolates from Madagascar and an additional 28 SNPs that differed between CO92 and all *Y. pestis* isolates from Madagascar.

5. Design of *Y. pestis* DNA Capture and Enrichment System

The pan-genome of a set of 373 *Y. pestis* genomes was determined with LS-BSR v1.2.3 (*13*), resulting in 16,171 unique coding region sequences (CDSs). CDSs shorter than 120nts were filtered from the analysis, as well as any genes containing ambiguous nucleotide characters, leaving 13,290 CDSs. CDSs were sliced into 120nt fragments, overlapping by 60nts, resulting in 126,938 potential capture probes. These probes were clustered with USEARCH v11 (*14*) at 80% ID, resulting in 111,329 remaining probes. Probes were then screened against *Y. pestis* rRNA genes (5S, 16S, 23S) with LS-BSR, and probes with a blast score ratio (BSR) value >0.8 (*15*) to any rRNA gene were removed. Remaining probes were aligned back against the set of 373 *Y. pestis* genomes with LS-BSR and BSR values were calculated. Probes conserved only in a single *Y. pestis* genome (BSR value >0.8) were removed as they could represent contamination, resulting in a set of 107,048 potential probes. Remaining probes were screened with LS-BSR against a set of >125,000 non-*Y. pestis* bacterial genomes in GenBank and those with a BSR of >0.8 were removed. Because we enriched from human sputum samples, the probes also were aligned against the human genome and any probes with significant alignments were removed from the design, resulting in a final set of 99,353 probes that covered ~88.8% of the CO92 reference genome. Regions with extremely high GC content (>50% GC) or extremely low GC

content (<22%) are considered difficult to hybridize using probes. To increase the likelihood of capturing these regions, our library design included a boosting strategy wherein probes corresponding to these types of regions were multiplied by 2X-10X copies, assigning higher redundancy to the most extreme regions (>70% and <15% GC). The final set of probes was ordered from Agilent Technologies.

6. *Y. pestis* DNA Capture, Enrichment, and Sequencing from Select Sputum Samples

DNA extraction of clinical samples and selection process for enrichment. *Y. pestis* DNA capture, enrichment, and sequencing was attempted on 42 residual sputum samples (Appendix 1 Table 2). The original 42 sputum samples were obtained from cases categorized as probable or confirmed based upon the testing procedures detailed in Section 1 above. These 42 samples were selected for *Y. pestis* DNA capture and enrichment because they were obtained from the urban areas of Antananarivo or Toamasina, or were associated with the first urban transmission chain (Table 2). DNA was extracted from remaining residuals of these 42 sputum samples using the Qiagen QIAmp kit following manufacture protocol (Qiagen, Valencia, CA) and the resulting extracts were shipped from IPM to NAU. At NAU, an additional PCR was performed using a published *pla*-specific TaqMan assay (*16*); all 42 samples yielded positive results (Appendix 1 Table 2).

Library preparation. The complex DNA extracts (<1 ng-100 ng) from the 42 human residual sputum samples were sheared to ~250 bp using a QSonica Q800 Sonicator (QSonica, Newtown, CT) at 60% amplitude, with 15 sec on/off settings. Size of fragments was evaluated using a Fragment Analyzer (Agilent, Santa Clara, CA). Sheared samples were end-repaired, A-tailed, indexed with adaptor ligation, and amplified using the SureSelect XT-low input (LI) sample kit (Agilent Technologies, Santa Clara, CA). All DNA purification steps were carried out using Agencourt AMPure XP beads (0.8X bead ratio; Beckman Coulter, Brea, CA). Briefly, end repair and A-tailing of the sheared DNA were completed using the SureSelectXT-LI End Repair/A-tailing Reaction Mix (Agilent Technologies, Santa Clara, CA). A single index adaptor was ligated to the ends of the DNA fragments using the SureSelectXT Ligation Master Mix, followed by bead purification. Each ligated fragment was uniquely indexed through PCR-

amplification for 9 cycles (2 min at 98°C, 9 cycles for 30 s at 98°C, 30 s at 60°C, 1 min at 72°C, and a final extension of 5 min at 72°C), followed by bead purification. Final library size and quantity were assessed by Fragment Analyzer and Qubit Br dsDNA (ThermoFisher Scientific, Waltham, MA), respectively.

Probe-hybridization. To reduce the probability of capturing off-targets, each sample library was enriched in a single reaction instead of pooling multiple samples. Due to the ~50% GC content of the *Y. pestis* genome, the fast-hybridization protocol using hybridization reagents from SureSelect (Agilent Technologies, Santa Clara, CA) was used. Approximately 2000 ng of the total library was hybridized at 65°C for 3 hours (following manufacturer's protocol) with probes. Hybridized libraries were recovered using 50 uL Dynabeads MyOne Streptavidin T1 Kit (Thermo Fisher Scientific, Waltham, MA). To remove off target fragments, the beads underwent two different washing procedures according to the manufacturer's directions. PCR amplification occurred directly from washed beads with the SureSelect XT-LI Primer Mix (Agilent Technologies, Santa Clara, CA) using the same PCR conditions as the library preparation but with a 14-cycle parameter to increase the concentration of captured library. The amplicons were separated from the beads using a magnetic plate and transferred to a new tube. To amplify the residual capture library remaining on the beads, a second PCR was performed directly on the beads using KAPA HiFi PCR ready mix (Roche KAPA Biosystems, Wilmington, DE Cat# KK2612). The captured libraries from both PCR events were combined and purified. To further enrich for *Y. pestis* signal, we performed a second round of capture enrichment on the captured libraries following the same method as the first enrichment. Library quantity was assessed by Qubit Br dsDNA (Thermo Fisher Scientific, Waltham, MA). The size and quality were assessed by Fragment Analyzer.

Sequencing. Final libraries were quantified using the KAPA Library Quantification Kit (ROX Low, Roche KAPA Biosystems, Wilmington, DE) and pooled in equimolar concentrations. Sequencing of the 42 enriched libraries was performed using the Illumina NextSeq 550 platform with the 500/550 High Output KT v2.3 (300 cycles) and 2 × 150 bp read lengths or MiSeq V3 (600 cycles) and 2 × 300 bp read lengths (Illumina, San Diego, CA).

7. Analysis of Enriched *Y. pestis* DNA from Select Sputum Samples

In silico SNP analysis of enriched samples. Raw reads for the 42 enriched sputum samples (Appendix 1 Table 2) were aligned to the CO92 reference genome (GCF_000009065.1) with minimap2 v2.24 (*6*). PCR duplicates were removed with Samtools v1.6 (*17*) and SNPs were called with GATK v4.2.6 (*7*) (*7*); SNPs were compiled across all samples with NASP v1.2.0 (*9*). Specific SNP positions used to construct the maximum likelihood phylogeny (Figure 2 in the main text) were then manually queried from the resulting NASP matrix; novel *Y. pestis* SNPs that may have been present in these enriched samples were not called. Known SNPs were called if there was at least 3x depth of coverage and >80% agreement among the reads. Known SNPs were identified in seven of the 42 enriched samples; raw reads for these seven enriched samples are available at NCBI under BioProject PRJNA1018588. Coverage of the reference varied across the seven enriched samples with identified known SNPs (Appendix 1 Table 3).

Confirmation of SNP calls with TaqMan assays. To confirm the genotypes of the known SNPs from the maximum likelihood phylogeny that were called from the sequencing data generated from the seven analyzed sputum samples (Appendix 1 Table 3), six of those SNPs were incorporated into Dual Probe TaqMan real-time PCR canSNP assays (Appendix 1 Tables 4 and 5). Primers (Integrated DNA Technologies, San Diego, CA) and allele-specific TaqMan-minor groove binding (MGB) probes (Life Technologies, Applied Biosystems, Foster City, CA) for each assay were designed using Primer Express software (Applied Biosystems) and the CO92 *Y. pestis* reference genome. PCR conditions and probe concentration for different assays varied and are described in Appendix 1 Table 4.

PCR amplification per assay was carried out in 10 µL volume with either 1× TaqMan Universal PCR master mix (Life Technologies, Applied Biosystems, Foster City, CA) or an in-house custom master mix. The in-house custom master mix comprised 1x PCR buffer without $MgCl_2$, $MgCl_2$ 5 mM, 0.2 mM deoxynucleoside triphosphate, 0.4 units of platinum Taq DNA polymerase (Invitrogen, Carlsbad, CA), and 6.36 µL molecular grade water. Each assay contained 0.2 µM of probes (derived and ancestral allele-specific) and forward and reverse primers at 0.6 µM to achieve 10 µL total volume. Two negative controls per SNP assay and two allele positive controls (representing ancestral and derived SNP states) were included with each experiment. Ancestral allele templates were genomic DNA extracts from North American *Y.*

pestis isolates A1122 and CO92. Derived allele templates for all five TaqMan assays were synthetically designed and printed on Genomic-block gene fragment (Integrated DNA Technologies, San Diego, CA). Thermal cycling parameters were as follows: initial denaturation at 95°C for 5 min followed by 50 cycles of 95°C for 15 s and 60 - 63°C (Appendix 1 Table 4) for 60 sec. All PCR amplifications were performed with the Applied Biosystems QuantStudio Flex Real-Time PCR System (Invitrogen, ThermoFisher).

SNP genotyping data and interpretation. Partial *Y. pestis* SNP genotyping calls were made for 14 of the SNPs used to construct the maximum likelihood phylogeny (Figure 2 in the main text) in seven enriched sputum samples (Appendix 1 Table 5). Complete genotyping data were not available for these samples due to the poor quality of the sputum samples (see main text and Appendix 1 Table 2). For this same reason, genotyping data were not available for the other 35 enriched sputum samples. The SNP genotypes that were determined were based upon sequence data generated from enriched sputum samples, TaqMan assays performed on the enriched sequencing libraries, or both (Appendix 1 Table 5); in all cases there was agreement between TaqMan and sequencing data when both data types were generated for a particular SNP in a given sample.

Just one isolate, 17/17, was obtained from the transmission chain described in detail in the main text, the transmission chain associated with Emergence 2. SNP calls were made in four sputum samples collected from human cases known to be associated with this transmission chain and one additional sputum sample not previously known to be associated with this transmission chain (Appendix 1 Table 5). One SNP specific to isolate 17/17 in the maximum likelihood phylogeny and two SNPs specific to phylogenetic group “s” or a subclade within this group in the maximum likelihood phylogeny were identified in sputum sample 135–2017; this sample was obtained on 11 September 2017 from Case 4 in this transmission chain. Five SNPs specific to isolate 17/17 in the maximum likelihood phylogeny and seven SNPs specific to phylogenetic group “s” or a subclade within this group in the maximum likelihood phylogeny were identified in sputum sample 121–2017; this sample was obtained on 11 September 2017 from Case 7 in this transmission chain. Note that sputum sample 121–2017 from Case 7 was also the source of isolate 17/17 (see main text); all SNPs genotyped in the enrichment of this sputum sample were identical in the whole genome sequence of isolate 17/17, providing confidence in the overall enrichment approach. Five SNPs specific to isolate 17/17 in the maximum likelihood phylogeny

and seven SNPs specific to phylogenetic group “s” or a subclade within this group in the maximum likelihood phylogeny were identified in sputum sample 125–2017; this sample was obtained on 12 September 2017 from Case 15 in this transmission chain. One SNP specific to isolate 17/17 in the maximum likelihood phylogeny was identified in sputum sample 184–2017; this sample was obtained on 12 September 2017 from Case 22 in this transmission chain. Finally, three SNPs specific to isolate 17/17 in the maximum likelihood phylogeny were identified in sputum sample 2093–2017. This sample was obtained from an Antananarivo resident on 8 November 2017 with no recent travel outside the city and no known contact with the cases above, suggesting community spread of this transmission chain in Antananarivo and persistence there until at least November 2017.

A single derived SNP call was made from sputum sample 819–2017 (Appendix 1 Table 5), in a SNP that is specific to all of the “j” phylogenetic group isolates in the maximum likelihood phylogeny (Figure 2 in the main text). This sputum sample was collected on 12 October 2017 from a resident of Antananarivo with no recent travel history. Two separate emergence events were associated with the j phylogenetic group (Table 1 in main text): Emergence 8 (first onset date: 26 September 2017, see below) and Emergence 10 (first onset date: 3 October 2017, see below). There was documented travel to Antananarivo by a foreign tourist that presented at hospital in Antananarivo 1 October with PP symptoms; he had traveled outside Antananarivo, but the specific details are unknown. His sputum sample yielded isolate 35/17, which is closely related to multiple human isolates obtained from BP cases in Tsiroanomandidy District starting in September that are assigned to the j phylogenetic group (Figure 2 in the main text), suggesting plague activity in this rural focus as the ultimate source of the infection in the tourist, from whom no known secondary cases were reported. Thus, it is possible that the infection in the Antananarivo resident that yielded sputum sample 819–2017 was the result of additional, undocumented cases associated with this transmission chain in Antananarivo. Alternatively, Emergence 10 was also assigned to phylogenetic group j (Figure 2 in the main text), so it is also possible that the individual that yielded sputum sample 819–2017 could have been infected via a transmission chain from that other event that involved undocumented travel to Antananarivo. Finally, since the single SNP called in sputum sample 819–2017 is specific to all of the j phylogenetic group isolates in the maximum likelihood phylogeny (Figure 2 in the main text), the infection in this individual could have resulted from

yet another, undocumented emergence event associated with the j phylogenetic group. Regardless, this documents that a transmission chain associated with the j phylogenetic group was present in Antananarivo and it was distinct from the other transmission chains documented there, which were associated with Emergences 2, 7, and 12 (Table 1 in the main text).

A single derived SNP call was made from sputum sample 1494–2017 (Appendix 1 Table 5), in a SNP that is specific to human isolates 30/17, 32/17, 33/17, 34/17, 48/17, and 55/17 from the "β" phylogenetic group (Figure 2 in the main text, below); all of these isolates except isolate 34/17 were obtained in Ambalavao District as part of the investigation of Emergences 12 and 20 (see below). Sputum sample 1494–2017 was collected on 24 October 2017 from an Antananarivo resident with no travel history. Isolate 34/17 was reportedly collected from a newborn baby in Antananarivo 15 October; his parents were residents of Antananarivo with no recent travel. The baby was febrile and transferred to a children's hospital as a suspect plague case, where a bronchoscope was used to collect a sputum sample from him. The sputum sample attributed to this newborn yielded isolate 34/17, which is identical to isolates 30/17, 32/17, 33/17, and 55/17 (Figure 2 in the main text). No secondary cases were reported from the baby, but the SNP call in sputum sample 1494–2017 (Appendix 1 Table 5) suggests subsequent community spread of closely related *Y. pestis* in Antananarivo.

8. Detailed Information on Multiple Emergences of *Y. pestis* from Environmental Reservoirs into Humans

Human plague emerged independently from environmental reservoirs into humans at least 20 times in multiple rural endemic foci in Madagascar from August-November 2017 (Table 1 in the main text). These events occurred in 19 different communes located in 12 different districts in the central and northern highlands (Figure 1, panel A in the main text) and clinical *Y. pestis* isolates obtained from them were closely related to previous isolates from those locations (Figure 2 in the main text).

The following provides more information on the 20 emergence events. All dates are from 2017.

- Emergence 1

- Human BP isolate 14/17 assigned to phylogenetic subclade s04 within major phylogenetic group "s" in the phylogeny
- District/commune of origin: Miarinarivo/Ambatomanjaka
- BP isolates: 14/17 (from sample 110–2017)
- PP isolates: None
- Other cases: None
- Onset dates associated w/ these isolates/cases: 14/17 (110–2017): 13 August
- Travel: No
- Note: 14/17 is the earliest culture confirmed BP isolate from 2017 and the earliest 2017 isolate overall in our WGS phylogeny

- Emergence 2
  - Human primary PP isolate 17/17 assigned to phylogenetic subclade s13 within major phylogenetic group "s" in the phylogeny
  - District/commune of origin: Ankazobe/Marondry
  - BP isolates: None
  - PP isolates: 17/17 (121–2017)
  - Other cases: Yes – see Table 2 in the main text
  - Onset dates associated w/ these isolates/cases: 17/17 (121–2017): 6 September for this case (Case 7 in the main text), but onset date for Case 1 from this transmission chain (see main text) was 25 August
  - Travel: Yes – extensive. See Figure 1, panel B and Table 2 in main text.
- Emergence 3
  - Human BP isolates 15/17 and 19/17 assigned to phylogenetic subclade s03 within major phylogenetic group "s" in the phylogeny
  - District/commune of origin: Miarinarivo/Anosibe Ifanja
  - BP isolates: 15/17 (113–2017), 19/17 (114–2017)

  - PP isolates: None
  - Other cases: Yes – see notes
  - Onset dates associated w/ these isolates/cases: 19/17 (114–2017): 26 August; 15/17 (113–2017): 27 August
  - Travel: No
  - Notes: The individuals that yielded isolates 15/17 and 19/17 came from the same hamlet (Soanafindra), Fokontany Ambatolampy, Commune Anosibe Ifanja, and District Miarinarivo. Two other suspected cases were notified from the same location at almost the same time, but they did not yield isolates. These two other cases were also BP. They are probably all contacts of each other. Notification forms of all these cases mentioned the presence of dead rats either inside houses or in the village.
- Emergence 4
  - Human BP isolate 16/17 assigned to phylogenetic subclade q15 within major phylogenetic group "q" in the phylogeny
  - District/commune of origin: Moramanga/Ambohibary
  - BP isolates: 16/17 (117–2017)
  - PP isolates: None
  - Other cases: Yes – see notes
  - Onset dates associated w/ these isolates/cases: Contact #1 (see below): 2 September; Contact #2 (see below): 3 September; 16/17 (117–2017): 4 September
  - Travel: No
  - Notes: The individual that yielded isolate 16/17 had two other BP contacts, but there were no notification forms for these other contacts. These two contacts were found dead in the same kitchen with the individual that yielded

16/17. Contact 1: 13 yo, presence of sub-maxillary bubo. Contact 2: 6 yo, presence of sub-maxillary bubo.

- Emergence 5
  - Human BP isolates 18/17, 29/17, 31/17, and 40/17 assigned to phylogenetic subclade s04 within major phylogenetic group "s" in the phylogeny
  - District/commune of origin: Tsiroanomandidy/Tsinjoarivo Imanga (18/17, 31/17, 40/17) and Ambararatabe (29/17)
  - BP isolates: 18/17 (176–2017), 29/17 (724–2017), 31/17 (1228–2017), 40/17 (1875–2017)
  - PP isolates: None
  - Other cases: None
  - Onset dates associated w/ these isolates/cases: 18/17 (176–2017): 16 September; 29/17 (724–2017): 7 October; 31/17 (1228–2017): 12 October; 40/17 (1875–2017): 21 October
  - Travel: No
  - Notes: No relationship among these cases, except the individuals that yielded isolates 18/17, 31/17, and 40/17 all live in the same commune of Tsinjoarivo Imanga. Notification of dead rats on the form for the individual that yielded isolate 31/17.
- Emergence 6
  - Human BP isolates 27/17 and 50/17 assigned to phylogenetic subclade y03 within major phylogenetic group "y" in the phylogeny
  - District/commune of origin: Manandriana/Ambohimahazo (27/17), Ambositra/Ivato (50/17)
  - BP isolates: 27/17 (591–2017), 50/17 (2299–2017)
  - PP isolates: None
  - Other cases: None

- Onset dates associated w/ these isolates/cases: 27/17 (591–2017): 17 September; 50/17 (2299–2017): 12 November
- Travel: No

- Emergence 7
    - Human BP isolate 20/17 and primary PP isolate 21/17 assigned to phylogenetic subclade s14 within major phylogenetic group “s” in the phylogeny and are identical to each other (i.e., no SNP differences)
    - District/commune of origin: Andramasina/Sabotsy Ambohitromby
    - BP isolates: 20/17 (189–2017)
    - PP isolates: 21/17 (212–2017)
    - Other cases: None recorded – see notes
    - Onset dates associated w/ these isolates/cases: 20/17 (189–2017): 21 September; 21/17 (212–2017): 25 September
    - Travel: Yes, but unclear by whom. Isolate 21/17 was obtained from an individual in Antananarivo with no reported travel.
    - Notes: No known relationship between the individuals that yielded these two isolates and no reported travel by either of them. The individual that yielded 20/17 had BP that was progressing to SPP but also had no known travel. Clinical signs: 39.6°C, cervical bubo (bean size), cough <5 days; bubo sample collected on 22 September 2017. The likely explanation here is that this individual – or someone else infected from the same environmental source – initiated a PP outbreak in this district and then someone infected from that outbreak traveled to Antananarivo leading to a transmission chain there that infected the individual that yielded 21/17.
- Emergence 8
    - Human BP isolates 23/17, 24/17, 25/17, 41/17, 44/17, and 46/17 and primary PP isolate 35/17 assigned to phylogenetic subclade j02 within major phylogenetic group “j” in the phylogeny

- District/commune of origin: Tsiroanomandidy/Bemahatazana
- BP isolates: 23/17 (284–2017), 24/17 (283–2017), 25/17 (282–2017), 41/17 (722–2017), 44/17 (2098–2017), 46/17 (2097–2017)
- PP isolates: Yes. Isolate 35/17 (309–2017) differs by just one SNP from BP isolates 23/17, 25/17, and 41/17.
- Other cases: None
- Onset dates associated w/ these isolates/cases: 23/17 (284–2017): 26 September; 24/17 (283–2017): 30 September; 25/17 (282–2017): 30 September; 35/17 (309–2017): 1 October; 41/17 (722–2017): 6 October; 44/17 (2098–2017): 7 November; 46/17 (2097–2017): 7 November
- Travel: Yes. Isolate 35/17 was from a tourist who traveled around Madagascar, but the details of his travel are unknown. He was treated in Antananarivo, which is where isolate 35/17 was obtained from him.
- Notes: Multiple BP cases and multiple dates, so multiple emergences of BP from the environment in this location. BP cases arose for >1 month.

- Emergence 9
  - Human BP isolate 22/17 assigned to phylogenetic subclade s05 within major phylogenetic group “s” in the phylogeny
  - District/commune of origin: Manjakandriana/Ranovao
  - BP isolates: 22/17 (281–2017)
  - PP isolates: None
  - Other cases: None
  - Onset dates associated w/ these isolates/cases: 22/17 (281–2017): 2 October (probably not 100% accurate as she died the next day and this was BP)
  - Travel: Yes. The family of this individual took her to Antananarivo for treatment where she died on 3 October.
- Emergence 10

- Human BP isolate 45/17 assigned to phylogenetic subclade j02 within major phylogenetic group “j” in the phylogeny
- District/commune of origin: Tsiroanomandidy/Ambalanirana
- BP isolates: 45/17 (1230–2017)
- PP isolates: None
- Other cases: None
- Onset dates associated w/ these isolates/cases: 45/17 (1230–2017): 3 October
- Travel: No

- Emergence 11
  - Human primary PP isolate 28/17 assigned to phylogenetic subclade s03 within major phylogenetic group “s” in the phylogeny
  - District/commune of origin: Arivonimamo/Arivonimamo II
  - BP isolates: None
  - PP isolates: 28/17 (594–2017)
  - Other cases: None recorded – see notes
  - Onset dates associated w/ these isolates/cases: 28/17 (594–2017): 5 October
  - Travel: No
  - Notes: Obviously there were other cases associated with this one as it is a primary PP case, so it had to have been obtained from another individual with PP. But there is no information on those other cases.
- Emergence 12
  - Human BP isolates 30/17, 32/17, and 33/17 and primary PP isolates 34/17 and 55/17 assigned to major phylogenetic group “β” in the phylogeny and are identical to each other (i.e., no SNP differences)
  - District/commune of origin: Ambalavao/Miarinarivo
  - BP isolates: 30/17 (1099–2017), 32/17 (1095–2017), 33/17 (1097–2017)

- PP isolates: 34/17 (1024–2017) and 55/17 (2394–2017)
- Other cases: Yes – but did not yield isolates.
- Onset dates associated w/ these isolates/cases: 32/17 (1095–2017): 7 October; 1096–2017 (no isolate): 7 October; 33/17 (1097–2017): 8 October; 30/17 (1099–2017): 8 October; 34/17 (1024–2017): 14 October; 55/17 (2394–2017): 22 November
- Travel: Yes. Somehow the identical strain got from Ambalavao/Miarinarivo to Antananarivo (see notes).
- Notes: The individual that yielded isolate 32/17 had symptoms consistent with BP that progressed to secondary PP. Primary PP isolates 34/17 and 55/17 are both 100% identical to BP isolates 30/17, 32/17, and 33/17 in the phylogeny. Isolate 34/17 (onset date = 14 October) is reportedly from the newborn baby who was tested in Antananarivo (see main text). 55/17 (onset date = 22 November) is from an individual from Ambalavao/Miarinarivo with no known travel. So, this *Y. pestis* lineage was somehow transferred to Antananarivo but the details of this transfer are unknown.

- Emergence 13
  - Human BP isolate 39/17 assigned to phylogenetic subclade s05 within major phylogenetic group "s" in the phylogeny
  - District/commune of origin: Ankazobe/Fiadanana
  - BP isolates: 39/17 (1780–2017)
  - PP isolates: None
  - Other cases: None
  - Onset dates associated w/ these isolates/cases: 39/17 (1780–2017): 8 October
  - Travel: No
- Emergence 14

- Human BP isolate 38/17 assigned to major phylogenetic group "α" in the phylogeny
- District/commune of origin: Mandritsara/Andratamarina
- BP isolates: 38/17 (2095–2017)
- PP isolates: None
- Other cases: None
- Onset dates associated w/ these isolates/cases: 38/17 (2095–2017): 14 October
- Travel: No
- Note: This location is in the northern highlands.

- Emergence 15
  - Human BP isolate 37/17 assigned to phylogenetic subclade s03 within major phylogenetic group "s" in the phylogeny
  - District/commune of origin: Miarinarivo/Anosibe Ifanja
  - BP isolates: 37/17 (1542–2017)
  - PP isolates: None
  - Other cases: None
  - Onset dates associated w/ these isolates/cases: 37/17 (1542–2017): 17 October
  - Travel: No
- Emergence 16
  - Human BP isolate 49/17 assigned to phylogenetic subclade t11 within major phylogenetic group "t" in the phylogeny
  - District/commune of origin: Manandriana/Ambovombe Centre
  - BP isolates: 49/17 (2255–2017)
  - PP isolates: None
  - Other cases: None

  - Onset dates associated w/ these isolates/cases: 49/17 (2255–2017): 18 October
  - Travel: No
- Emergence 17
  - Human BP isolate 36/17 assigned to phylogenetic subclade x01 within major phylogenetic group "x" in the phylogeny
  - District/commune of origin: Arivonimamo/Mahatsinjo Est
  - BP isolates: 36/17 (1700–2017)
  - PP isolates: None
  - Other cases: None
  - Onset dates associated w/ these isolates/cases: 36/17 (1700–2017): 23 October
  - Travel: No
- Emergence 18
  - Human primary PP isolate 43/17 assigned to phylogenetic subclade q04 within major phylogenetic group "q" in the phylogeny
  - District/commune of origin of infection (see below): Antananarivo-Avaradrano/Manandriana
  - BP isolates: None
  - PP isolates: 43/17 (2048–2017)
  - Other cases: Yes – see notes
  - Onset dates associated w/ these isolates/cases: 43/17 (2048–2017): 30 October
  - Travel: Yes – see notes
  - Notes: The individual that yielded isolate 43/17 lived in Andramasina/Tankafatra but traveled to Antananarivo Avaradrano/Manandriana (Imerimandroso) before becoming sick. He had a contact at Imerimandroso who had a cough <5 days and who also died. This contact was not evaluated but mentioned in the notification form for the

individual that yielded 43/17. Thus, the environmental source of this isolate is listed as Antananarivo-Avaradrano/Manandriana, not Andramasina/Tankafatra, as no other cases were reported from Andramasina/Tankafatra.

- Emergence 19
  - Human BP isolate 47/17 assigned to phylogenetic subclade s03 within major phylogenetic group "s" in the phylogeny
  - District/commune of origin: Faratsiho/Antsampanimahazo
  - BP isolates: 47/17 (2154–2017)
  - PP isolates: None
  - Other cases: None
  - Onset dates associated w/ these isolates/cases: 47/17 (2154–2017): 7 November
  - Travel: No
- Emergence 20
  - Human BP isolate 48/17 assigned to major phylogenetic group "β" in the phylogeny
  - District/commune of origin: Ambalavao/Anjoma
  - BP isolates: 48/17 (2167–2017)
  - PP isolates: None
  - Other cases: None
  - Onset dates associated w/ these isolates/cases: 48/17 (2167–2017): 9 November
  - Travel: No
  - Note: Dead rats were present in the house of the individual that yielded this isolate.

References

1. Chanteau S, Rahalison L, Ralafiarisoa L, Foulon J, Ratsitorahina M, Ratsifasoamanana L, et al. Development and testing of a rapid diagnostic test for bubonic and pneumonic plague. Lancet. 2003;361:211–6. PubMed https://doi.org/10.1016/S0140-6736(03)12270-2

2. Randremanana R, Andrianaivoarimanana V, Nikolay B, Ramasindrazana B, Paireau J, Ten Bosch QA, et al. Epidemiological characteristics of an urban plague epidemic in Madagascar, August-November, 2017: an outbreak report. Lancet Infect Dis. 2019;19:537–45. PubMed https://doi.org/10.1016/S1473-3099(18)30730-8

3. Rasoamanana B, Rahalison L, Raharimanana C, Chanteau S. Comparison of Yersinia CIN agar and mouse inoculation assay for the diagnosis of plague. Trans R Soc Trop Med Hyg. 1996;90:651. PubMed https://doi.org/10.1016/S0035-9203(96)90420-4

4. Andrianaivoarimanana V, Wagner DM, Birdsell DN, Nikolay B, Rakotoarimanana F, Randriantseheno LN, et al. Transmission of Antimicrobial Resistant Yersinia pestis During a Pneumonic Plague Outbreak. Clin Infect Dis. 2021. PubMed

5. Parkhill J, Wren BW, Thomson NR, Titball RW, Holden MT, Prentice MB, et al. Genome sequence of Yersinia pestis, the causative agent of plague. Nature. 2001;413:523–7. PubMed https://doi.org/10.1038/35097083

6. Li H. Minimap2: pairwise alignment for nucleotide sequences. Bioinformatics. 2018;34:3094–100. PubMed https://doi.org/10.1093/bioinformatics/bty191

7. McKenna A, Hanna M, Banks E, Sivachenko A, Cibulskis K, Kernytsky A, et al. The Genome Analysis Toolkit: a MapReduce framework for analyzing next-generation DNA sequencing data. Genome Res. 2010;20:1297–303. PubMed https://doi.org/10.1101/gr.107524.110

8. Delcher AL, Salzberg SL, Phillippy AM. Using MUMmer to identify similar regions in large sequence sets. Curr Protoc Bioinformatics. 2003 Feb;Chapter 10:Unit 10 3.

9. Sahl JW, Lemmer D, Travis J, Schupp JM, Gillece JD, Aziz M, et al. NASP: an accurate, rapid method for the identification of SNPs in WGS datasets that supports flexible input and output formats. Microb Genom. 2016;2:e000074. PubMed https://doi.org/10.1099/mgen.0.000074

10. Benson G. Tandem repeats finder: a program to analyze DNA sequences. Nucleic Acids Res. 1999;27:573–80. PubMed https://doi.org/10.1093/nar/27.2.573

11. Minh BQ, Schmidt HA, Chernomor O, Schrempf D, Woodhams MD, von Haeseler A, et al. IQ-TREE 2: New Models and Efficient Methods for Phylogenetic Inference in the Genomic Era. Mol Biol Evol. 2020;37:1530–4. PubMed https://doi.org/10.1093/molbev/msaa015

12. Kalyaanamoorthy S, Minh BQ, Wong TKF, von Haeseler A, Jermiin LS. ModelFinder: fast model selection for accurate phylogenetic estimates. Nat Methods. 2017;14:587–9. PubMed https://doi.org/10.1038/nmeth.4285

13. Sahl JW, Caporaso JG, Rasko DA, Keim P. The large-scale blast score ratio (LS-BSR) pipeline: a method to rapidly compare genetic content between bacterial genomes. PeerJ. 2014;2:e332. PubMed https://doi.org/10.7717/peerj.332

14. Edgar RC. Search and clustering orders of magnitude faster than BLAST. Bioinformatics. 2010;26:2460–1. PubMed https://doi.org/10.1093/bioinformatics/btq461

15. Rasko DA, Myers GS, Ravel J. Visualization of comparative genomic analyses by BLAST score ratio. BMC Bioinformatics. 2005;6:2. PubMed https://doi.org/10.1186/1471-2105-6-2

16. Iqbal SS, Chambers JP, Goode MT, Valdes JJ, Brubaker RR. Detection of Yersinia pestis by pesticin fluorogenic probe-coupled PCR. Mol Cell Probes. 2000;14:109–14. PubMed https://doi.org/10.1006/mcpr.2000.0295

17. Li H, Handsaker B, Wysoker A, Fennell T, Ruan J, Homer N, et al.; 1000 Genome Project Data Processing Subgroup. The Sequence Alignment/Map format and SAMtools. Bioinformatics. 2009;25:2078–9. PubMed https://doi.org/10.1093/bioinformatics/btp352

Appendix Table 1. 91 *Y. pestis* whole genomes sequences used to construct the maximum likelihood phylogeny*

Strain	Accession#	Year	Source	District	Commune	Lineage
20/17	SRS4032725	2017	Human	Andramasina	Sabotsy Ambohitromby	s14
21/17	SRS4032726	2017	Human	Antananarivo Avaradrano	Ankadikely Ilafy	s14
56/13	SRR11676332	2013	Human	Faratsiho	Faratsiho	s14
160/07	SRS4032764	2007	Human	Faratsiho	Ramainandro	s15
42/08	SRR4175426	2008	Human	Faratsiho	Faratsiho	s16
78/06	SRR4175415	2006	Rat	Betafo	Ambohimanambola	s13
17/17	SRS4032722	2017	Human	Mahajanga I	Mahabibo	s13
207/11	SRS4032760	2011	Human	Faratsiho	Ramainandro	s13
65/16	SRR12199758	2016	Rat	Ankazobe	Ambohitromby	s13
25/09	SRS4032763	2009	Human	Faratsiho	Ramainandro	s13
178/12	SRS4032749	2012	Human	Ankazobe	Marondry	s13
67/05	SRS4032748	2005	Human	Faratsiho	Ramainandro	s13
24/14	SRR12199765	2014	Human	Ankazobe	Ambohitromby	s05
155/13	SRS4032767	2013	Human	Ankazobe	Marondry	s05
174/11	SRS4032751	2011	Human	Ankazobe	Marondry	s05
214/08	SRR12199763	2008	Human	Ankazobe	Ambohitromby	s05
25/16	SRR12199759	2016	Human	Ankazobe	Ambohitromby	s05
13/14	SRR12199764	2014	Human	Ankazobe	Ambohitromby	s05
146/14	SRR12199760	2014	Rat	Ankazobe	Ambohitromby	s05
39/17	SRS4032758	2017	Human	Ankazobe	Fiadanana	s05
79/15	SRS4032770	2015	Human	Ankazobe	Marondry	s05
02/09	SRR4175439	2009	Human	Manjakandriana	Ambatomena	s22
154/98B	SRR651089	1998	Human	Mahajanga I	Mahabibo	s09
296/03	SRR12199766	2003	Rat	Ankazobe	Ambohitromby	s05
324/04	SRR12199761	2004	Human	Ankazobe	Ambohitromby	s05
409/04	SRR12199762	2004	Human	Ankazobe	Ambohitromby	s05
193/07	SRR4175437	2007	Human	Manjakandriana	Ambatolaona	s17
22/17	SRS4032729	2017	Human	Manjakandriana	Ranovao	s05
18/17	SRS4032727	2017	Human	Tsiroanomandidy	Tsinjoarivo Imanga	s04
29/17	SRS4032737	2017	Human	Tsiroanomandidy	Ambararatabe	s04
31/17	SRS4032738	2017	Human	Tsiroanomandidy	Tsinjoarivo Imanga	s04
40/17	SRS4032761	2017	Human	Tsiroanomandidy	Tsinjoarivo Imanga	s04
14/17	SRS4032723	2017	Human	Miarinarivo	Ambatomanjaka	s04
15/17	SRS4032724	2017	Human	Miarinarivo	Anosibe Ifanja	s03
19/17	SRS4032728	2017	Human	Miarinarivo	Anosibe Ifanja	s03
37/17	SRS4032755	2017	Human	Miarinarivo	Anosibe Ifanja	s03
89/14	SRS4032766	2014	Rat	Mahajanga I	Mahajanga	s03
28/17	SRS4032735	2017	Human	Arivonimamo	Arivonimamo II	s03
47/17	SRS4032762	2017	Human	Faratsiho	Antsampanimahazo	s03
24/08	SRR12199767	2008	Human	Ankazobe	Ambohitromby	s03
39/07	SRR4175414	2007	Flea	Betafo	Inanantonana	s12
35/15	SRS4032765	2015	Human	Moramanga	Ampasimpotsy Gara	q12
139/06	SRR4175441	2006	Human	Betafo	Anosiarivo Manapa	q13
01/08	SRR4175443	2008	Human	Manjakandriana	Ranovao	q06
43/17	SRS4032753	2017	Human	Antananarivo Avaradrano	Manandriana	q04
16/17	SRS4032721	2017	Human	Moramanga	Ambohibary	q15
11/09	SRR4175442	2009	Human	Moramanga	Ambohibary	q14
135/11	SRR4175440	2011	Human	Moramanga	Lakato	q11
172/03	SRR4175416	2003	Human	Soavinandriana	Tamponala	j06
357/04	SRR4175417	2004	Human	Mandoto	Mandoto	j04
74/98	SRS4032745	1998	Rat	Brickaville	Ranomafana Est	j07
88/11	SRR4175419	2011	Human	Mandoto	Antanambao Ambary	j10
43/07	SRR4175418	2007	Flea	Betafo	Inanantonana	j02
45/17	SRS4032741	2017	Human	Tsiroanomandidy	Ambalanirana	j02
24/17	SRS4032731	2017	Human	Tsiroanomandidy	Bemahatazana	j02
41/17	SRS4032759	2017	Human	Tsiroanomandidy	Bemahatazana	j02
23/17	SRS4032730	2017	Human	Tsiroanomandidy	Bemahatazana	j02
25/17	SRS4032732	2017	Human	Tsiroanomandidy	Ambatolampy	j02
35/17	SRS4032752	2017	Human	Antananarivo Renivohitra	1er Arrondissement	j02
46/17	SRS4032742	2017	Human	Tsiroanomandidy	Bemahatazana	j02
44/17	SRS4032750	2017	Human	Tsiroanomandidy	Bemahatazana	j02
IP275	GCA_000168235	1995	Human	Ambalavao	Besoa	l02
48/17	SRS4032769	2017	Human	Ambalavao	Anjoma	β
32/17	SRS4032739	2017	Human	Ambalavao	Miarinarivo	β
55/17	SRS4032743	2017	Human	Ambalavao	Miarinarivo	β
34/17	SRS4032754	2017	Human	Antananarivo Avaradrano	Ankadikely Ilafy	β
30/17	SRS4032736	2017	Human	Ambalavao	Miarinarivo	β
33/17	SRS4032740	2017	Human	Ambalavao	Miarinarivo	β

Strain	Accession#	Year	Source	District	Commune	Lineage
201/07	SRR4175444	2007	Human	Ambatofinandrahana	Ambatofinandrahana	y05
27/17	SRS4032734	2017	Human	Manandriana	Ambohimahazo	y03
50/17	SRS4032746	2017	Human	Ambositra	Ivato	y03
62/09	SRR4175420	2009	Human	Antanifotsy	Ambohitompoina	z06
38/17	SRS4032757	2017	Human	Mandritsara	Andratamarina	α
55/08	SRR4175435	2008	Human	Antsirabe II	Tsarahonenana Sahanivotry	w02
67/09	SRR4175423	2009	Human	Antsirabe II	Manandona	t02
49/17	SRS4032744	2017	Human	Manandriana	Ambovombe Centre	t11
45/04	SRR4175421	2004	Human	Antsirabe II	Manandona	t11
12/09	SRR4175436	2009	Human	Fandriana	Miarinavaratra	u03
145/08	SRR4175428	2008	Human	Ambatofinandrahana	Soavina	h04
18/06	SRR4175425	2006	Human	Antsirabe II	Mangarano	h10
46/12	SRR4175429	2012	Human	Betafo	Alakamisy Marososona	h06
214/07	SRR4175427	2007	Human	Antsirabe II	Ambohitsimanova	h16
36/17	SRS4032756	2017	Human	Arivonimamo	Mahatsinjo Est	x01
24/09	SRR4175438	2009	Human	Faratsiho	Ramainandro	x03
102/06	SRS4032747	2006	Human	Faratsiho	Ramainandro	x03
09/12	SRR4175434	2012	Human	Betafo	Mahaiza	v03
30/10	SRR4175432	2010	Human	Antsirabe II	Ambohitsimanova	v06
95/07	SRR4175431	2007	Human	Betafo	Manohisoa	v13
197/11	SRR4175433	2011	Human	Betafo	Ambohimanambola	v09
69/04	SRR4175430	2004	Human	Mandoto	Ankazomiriotra	v10
CO92 (root)	GCA_001293415.1	1992	Human	N/A	N/A	N/A

*Sequences are ordered as they appear in the phylogeny (Figure 2 in main text) from top to bottom.

Appendix Table 2. Residual sputum samples from 42 probable or confirmed cases used for *Y. pestis* DNA capture, enrichment, and sequencing*

Sputum sample	District	Collection date	RDT result	Culture result	PCR (IPM) result	Case classification	PCR (NAU) result (Ct)
121–2017	Mahajanga I	14-Sep-17	POS	POS	POS	Confirmed	27.3
135–2017	Toamasina I	15-Sep-17	POS	NEG	POS	Confirmed	35.0
125–2017	Faratsiho	16-Sep-17	POS	NEG	POS	Confirmed	29.6
133–2017	Faratsiho	16-Sep-17	POS	NEG	POS	Confirmed	38.8
129–2017	Faratsiho	16-Sep-17	POS	NEG	NEG	Probable	37.7
184–2017	Vohemar	22-Sep-17	POS	NEG	NEG	Probable	38.4
597–2017	Antananarivo Renivohitra	10-Oct-17	NEG	-	POS	Probable	37.0
630–2017	Toamasina I	11-Oct-17	POS	NEG	NEG	Probable	38.3
647–2017	Toamasina I	11-Oct-17	POS	NEG	NEG	Probable	39.8
653–2017	Toamasina I	11-Oct-17	POS	NEG	NEG	Probable	37.2
663–2017	Toamasina I	11-Oct-17	POS	NEG	NEG	Probable	46.5
632–2017	Toamasina I	11-Oct-17	POS	NEG	NEG	Probable	38.9
636–2017	Toamasina I	11-Oct-17	POS	NEG	NEG	Probable	39.3
641–2017	Toamasina I	11-Oct-17	POS	NEG	NEG	Probable	38.5
666–2017	Toamasina I	11-Oct-17	POS	NEG	NEG	Probable	36.8
819–2017	Antananarivo Renivohitra	12-Oct-17	POS	NEG	NEG	Probable	40.8
841–2017	Antananarivo Renivohitra	12-Oct-17	POS	NEG	NEG	Probable	39.0
843–2017	Antananarivo Renivohitra	12-Oct-17	POS	NEG	NEG	Probable	37.2
1100–2017	Antananarivo Renivohitra	16-Oct-17	POS	NEG	POS	Confirmed	36.1
1074–2017	Antananarivo Renivohitra	16-Oct-17	POS	NEG	NEG	Probable	39.7
1078–2017	Antananarivo Atsimondrano	16-Oct-17	POS	NEG	NEG	Probable	38.5
1115–2017	Antananarivo Renivohitra	16-Oct-17	POS	NEG	NEG	Probable	37.8
1002–2017	Antananarivo Renivohitra	16-Oct-17	POS	NEG	NEG	Probable	37.9
1006–2017	Antananarivo Renivohitra	16-Oct-17	POS	NEG	NEG	Probable	39.4
1015–2017	Antananarivo Renivohitra	16-Oct-17	POS	NEG	NEG	Probable	38.8
1028–2017	Antananarivo Renivohitra	16-Oct-17	POS	NEG	NEG	Probable	38.6
1040–2017	Antananarivo Renivohitra	16-Oct-17	POS	NEG	NEG	Probable	37.7
1164–2017	Antananarivo Renivohitra	17-Oct-17	POS	NEG	NEG	Probable	37.6
1147–2017	Antananarivo Renivohitra	17-Oct-17	POS	NEG	NEG	Probable	38.4
1237–2017	Antananarivo Renivohitra	18-Oct-17	POS	NEG	NEG	Probable	37.9
1307–2017	Antananarivo Renivohitra	20-Oct-17	POS	NEG	NEG	Probable	37.3
1470–2017	Antananarivo Renivohitra	23-Oct-17	POS	NEG	NEG	Probable	37.7
1468–2017	Antananarivo Renivohitra	23-Oct-17	POS	NEG	NEG	Probable	38.0
1494–2017	Antananarivo Renivohitra	24-Oct-17	NEG	-	POS	Probable	36.3
1633–2017	Antananarivo Renivohitra	26-Oct-17	POS	NEG	NEG	Probable	38.2
1659–2017	Antananarivo Renivohitra	26-Oct-17	POS	NEG	NEG	Probable	39.7

Sputum sample	District	Collection date	RDT result	Culture result	PCR (IPM) result	Case classification	PCR (NAU) result (Ct)
1661–2017	Antananarivo Renivohitra	26-Oct-17	POS	NEG	NEG	Probable	48.5
1686–2017	Antananarivo Renivohitra	27-Oct-17	POS	NEG	POS	Confirmed	38.9
1762–2017	Antananarivo Renivohitra	30-Oct-17	POS	NEG	NEG	Probable	48.3
1866–2017	Antananarivo Renivohitra	2-Nov-17	POS	NEG	NEG	Probable	38.3
2093–2017	Antananarivo Renivohitra	10-Nov-17	NEG	-	POS	Probable	39.1
2152–2017	Antananarivo Renivohitra	15-Nov-17	POS	NEG	POS	Confirmed	38.2

*RDT, culture, and initial PCR results were performed on the original sputum samples as detailed in Section 1 above.

Appendix Table 3. Percent coverage of *Y. pestis* sequencing reads generated from seven enriched samples across the four genomic components of the CO92 reference

Enriched sample	CO92 chromosome (NC_003143.1)	CO92 plasmid pMT1 (AL117211.1)	CO92 plasmid pCD1 (AL117189.1)	CO92 plasmid pPCP1 (AL109969.1)
121–2017	96.11	40.86	97.97	30.23
125–2017	95.49	41.69	98.37	32.92
135–2017	16.20	10.81	46.80	11.97
1494–2017	6.18	3.10	7.75	5.08
184–2017	8.65	5.60	12.56	17.27
2093–2017	4.26	1.66	5.45	4.44
819–2017	8.34	4.02	12.98	5.77

Appendix Table 4. Primers, TaqMan®-MGB probes, and annealing temperatures for *Y. pestis* SNP genotyping assays*

SNP position in CO92 reference	Primer sequences (5′ to 3′)	Probe sequences (5′ to 3′)	Annealing temperature
3490108	F-GATAGGCGTAAGGCATTAAATAATGAC	D-6FAM-CCATAAGCGACACCAT	63
	R-GGTTTATATGCATTCGCTGGTCATTATT	A-VIC-CCATAAGCGGCACCA	
2297224	F-TCATACCATCAAGGACGACTGC	D-6FAM-CTAACATCGTCATTGAA	60
	R-TTTGTGCTGAACTGGGCGA	A-VIC-TAACATCGCCATTGAA	
2275118	F-GTTTGGCGGAACAAGGCTATA	D-6FAM-GTATCAAGGCGTGAGTG	60
	R-CGTGAATATCTTCAATGCGGTAA	A-VIC-TATCAAGGCGCGAGTG	
2524044	F-CAATATGGCCATAGGCAGCG	D-6FAM-CCATGGAGTTGCCA	60
	R-GCTGTTACCGGATTTGTCCGT	A-VIC-CCATGGTGTTGCCA	
2569128	F-GCCGTGCCAAGCGCTATC	*D-6FAM-CTGGCTAAAATTGGTGATG	60
	R-AGGCCTTGCCCAGCACTTC	*A-VIC-TCTGGCTAAAATCGGTGAT	
3289728	F-ACACTTGGAAGAATAACTCATGTACATCAG	D-6FAM-TCGATTTCATCTTACAACAC	60
	R-CTGTGCGCCGTAAGCCATAT	A-VIC-TCGATTTCATCTAACAACAC	

*Annealing temperatures are in C°. F: forward primer; R: reverse primer; D: probe for derived SNP state; A: probe for ancestral SNP state; Red text in probe sequence: SNP state; *: probe designed on the reverse complement

Appendix Table 5. *Y. pestis* SNP genotyping calls in seven enriched sputum samples*

SNP position in CO92	TaqMan assay?	CO92 allele (outgroup, ancestral state)	Sputum 135–2017 (Case 4)	Sputum 121–2017 (Case 7; isolate 17/17)	Sputum 125–2017 (Case 15)	Sputum 184–2017 (Case 22)	Sputum 2093–2017	Sputum 819–2017	Sputum 1494–2017	SNP specificity in the phylogeny
812303	NO	A		G (S)	G (S)					S group SNP found in isolate 17/17 and multiple other 2017 isolates and older isolates
2681067	NO	A		G (S)	G (S)					S group SNP found in isolate 17/17 and multiple other 2017 isolates and older isolates
3490108	YES	G		A (S/T)	A (S)					S group SNP found in isolate 17/17 and multiple other 2017 isolates and older isolates
4545787	NO	T		A (S)	A (S)					S group SNP found in isolate 17/17 and multiple other 2017 isolates and older isolates
1902978	NO	G	A (S)	A (S)	A (S)					S group SNP found in isolate 17/17 and multiple other 2017 isolates and older isolates
2274467	NO	G		A (S)	A (S)					S group SNP found in isolate 17/17 and multiple other 2017 isolates and older isolates
1539852	NO	C	T (S)	T (S)	T (S)					S group SNP found in isolate 17/17 and some older isolates but no other 2017 isolates
2297224	YES	C	T (S/T)	T (S/T)	T (S/T)	T (S)	T (T)			SNP on branch unique to 17/17 in WGS phylogeny
2275118	YES	C		T (S/T)	T (S/T)					SNP on branch unique to 17/17 in WGS phylogeny
2524044	YES	T		A (S/T)	A (S/T)		A (T)	T (S/T)		SNP on branch unique to 17/17 in WGS phylogeny
2569128	YES	G		A (S/T)	A (S/T)					SNP on branch unique to 17/17 in WGS phylogeny
3289728	YES	A		T (S/T)	T (S/T)		T (T)			SNP on branch unique to 17/17 in WGS phylogeny
420544	NO	C		C (S)	C (S)			T (S)		J group SNP found in all J group isolates in the phylogeny, including multiple 2017 isolates
2383628	NO	T		T (S)	T (S)				C (S)	β group SNP found in 2017 isolates 30/17,

SNP position in CO92	TaqMan assay?	CO92 allele (outgroup, ancestral state)	Sputum 135–2017 (Case 4)	Sputum 121–2017 (Case 7; isolate 17/17)	Sputum 125–2017 (Case 15)	Sputum 184–2017 (Case 22)	Sputum 2093–2017	Sputum 819–2017	Sputum 1494–2017	SNP specificity in the phylogeny
										32/17, 33/17, 34/17, 48/17, and 55/17

*Colors in the first row and first and last columns correspond to the colors assigned to specific phylogenetic groups in the maximum likelihood phylogeny (Figure 2 in the main text). Blue and yellow colors indicated ancestral and derived state of SNPs, respectively. (S): SNP call from sequencing data only; (T): SNP call from TaqMan data only; (S/T): SNP call from both sequencing and TaqMan data.
